# Illness anxiety disorder: A qualitative study of people with health anxiety and their experiences seeking and avoiding medical care

**DOI:** 10.1111/bjc.70005

**Published:** 2025-07-08

**Authors:** Katarina Kikas, Aliza Werner‐Seidler, Brittany Corkish, Emily Upton, Monique Holden, Jill M. Newby

**Affiliations:** ^1^ Black Dog Institute University of New South Wales Sydney New South Wales Australia; ^2^ School of Psychology University of New South Wales Sydney Australia

**Keywords:** diagnostic criteria, health anxiety, healthcare, help‐seeking, illness anxiety disorder, somatoform disorder

## Abstract

**Objective:**

Illness anxiety disorder (IAD) has two subtypes in the DSM‐5: ‘care‐seeking’ and ‘care‐avoidant’, with a third subtype, ‘care fluctuating’ identified in previous research. This study explores the experiences of individuals with IAD when seeking and avoiding medical care.

**Design and Methods:**

We recruited self‐identified health‐anxious individuals via online social media advertisements. Participants completed a demographic questionnaire and a diagnostic interview assessing IAD and comorbidities. Participants meeting IAD criteria (*N* = 37, mean age = 39, 76% female) completed a qualitative interview about their experiences seeking and avoiding medical care, analyzed using reflexive thematic analysis.

**Results:**

Participants reported that internal and external factors influenced help‐seeking behaviours. Internal factors were physical symptoms, worry, fear, reassurance‐seeking, and the motivation to stay healthy for their children. External factors included past experiences with health professionals, cost of care, and a busy lifestyle. Worry about missing a serious disease, symptom severity, and emotional fatigue from repeated care‐seeking contributed to fluctuations in help‐seeking. Negative past experiences with health professionals also impacted willingness to seek medical care.

**Conclusions:**

Reasons behind different help‐seeking behaviours in IAD are complex. This study provides insight into the help‐seeking experiences of individuals with IAD and the factors influencing these behaviours, which can inform targeted treatment approaches for IAD.

**Practitioner points**

- DSM‐5 illness anxiety disorder care‐seeking and care‐avoiding subtypes have not been empirically examined, as well as a third subtype: ‘care‐fluctuating’, identified in past research.
- Care‐seeking was influenced by physical symptoms, worry, past negative experiences, and reassurance‐seeking.
- Care‐avoidance was driven by cost, busy lifestyle, diagnosis fear, and not being taken seriously.
- Symptom severity, missed disease worry, and emotional fatigue drive fluctuations in care‐seeking and avoidance.

## INTRODUCTION

Illness anxiety disorder (IAD) is a newly recognized disorder in the DSM‐5, which describes individuals who frequently and excessively worry about their health (American Psychiatric Association [APA], 2013). Individuals with IAD experience significant fear of having or developing a serious, undiagnosed illness, and engage in maladaptive cognitive and behavioural processes, such as rumination, excessive reassurance seeking, and frequent body checking. IAD was introduced in the DSM‐5 to capture health anxious individuals who have mild or no physical symptoms, whereas health anxious individuals with moderate to severe physical symptoms are captured by somatic symptom disorder (APA, 2013). Living with illness anxiety places a significant strain on individuals' lives, leading to more sick leave than the general population (Eilenberg et al., 2015), greater distress in personal relationships (Barsky et al., 1998), and disruptions to daily activities, such as household duties (Sunderland et al., 2013). The current prevalence rates of IAD remain unknown, likely due to its recent introduction in the DSM‐5. However, severe levels of health anxiety, more broadly, are highly prevalent within the general population, with estimates of lifetime prevalence being 5.7% in the Australian population (Sunderland et al., 2013). Given that individuals with health anxiety often seek excessive medical reassurance, it is unsurprizing that the prevalence of severe health anxiety within medical settings is even higher than in the general population, estimated to range between 7% and 19.9% (Pandey et al., 2017; Tyrer et al., 2019).

The DSM‐5 categorizes individuals with IAD into two subtypes: ‘care‐seeking’ and ‘care‐avoidant’. Individuals who seek excessive medical care fall into the ‘care‐seeking’ subtype, while those who avoid medical care fall into the ‘care‐avoidant’ subtype. Although these subtypes seem to offer a useful way to address the heterogeneity in IAD, few studies have examined them in‐depth (Kikas et al., 2024). To date, the only study that has explored DSM‐5 IAD subtypes investigated their prevalence in a sample of participants signing up to participate in a treatment trial (Newby et al., 2017). In this study, participants were asked to self‐identify as ‘care‐seeking’, ‘care‐avoidant’, or as a third ‘fluctuating’ subtype, added by the authors, for individuals who alternate between seeking and avoiding care. The findings revealed that the care‐avoidant subtype was least prevalent, followed by the care‐seeking subtype, while the fluctuating subtype was most common. However, there is no research on why individuals with IAD engage in these behaviours.

Exploring the reasons behind why individuals with IAD seek, avoid, or fluctuate between seeking and avoiding medical care is important knowledge for clinicians and researchers. This is important because safety behaviours (i.e., behaviours performed with the intention to reduce perceived health threats and protect one's health), avoidance of health‐related information and situational triggers (i.e., medical settings), and medical reassurance‐seeking (i.e., repeated medical requests aimed at reducing concern about having a serious illness) have been shown to exacerbate health anxiety over time (Abramowitz & Moore, 2007; Meyer et al., 2023; Olatunji et al., 2011; Warwick & Salkovskis, 1990). Further, excessively seeking medical care is not only time‐consuming for individuals but also costly to the healthcare system. Studies show higher healthcare utilization in individuals with health anxiety compared to those with diagnosed medical conditions but no health anxiety (Fink et al., 2010). Conversely, avoiding medical care can lead to prolonged health problems and delayed detection of serious diseases (Garbuz et al., 2006; Lund‐Nielsen et al., 2011). When medical care is eventually sought, it often requires more intensive treatment, resulting in significant costs to the healthcare system (Byrne, 2008). Therefore, understanding the factors influencing these behaviours is essential for developing targeted treatment options to ultimately reduce the burden of illness anxiety on both the individual and society.

Researchers have proposed hypotheses to explain these behaviours in illness anxiety. For example, it has been hypothesized that individuals with IAD avoid care due to an overwhelming fear of being diagnosed with their feared illness and excessively seek care out of a need for reassurance (Tyrer, 2018; Tyrer et al., 2016). However, these hypotheses have not been empirically tested, leaving research in this area limited. In contrast, studies on the general population have explored reasons individuals avoid care. Taber et al. (2015) identified three barriers that prevent individuals from seeking medical care: (i) traditional barriers, such as cost, lack of time, or lack of health insurance; (ii) physician‐related barriers, including negative past experiences with health professionals; and (iii) affective concerns, such as fear of bad news or embarrassment. Other studies have also highlighted cost and time constraints as significant barriers to receiving healthcare (Corscadden et al., 2017; Smith et al., 2018; Sulku et al., 2023). However, these barriers have not been investigated in individuals who excessively worry about their health or those diagnosed with IAD.

Although research on care avoidance in individuals with illness anxiety is lacking, two studies have investigated what motivates individuals with a DSM‐IV diagnosis of hypochondriasis to seek reassurance, such as through medical care (Halldorsson & Salkovskis, 2017; Okita et al., 2016). A study by Okita et al. (2016) qualitatively explored reassurance‐seeking across various domains, including the internet, TV, magazines, close networks such as friends and family, and health professionals in ten individuals with hypochondriasis. The authors found that health anxious individuals specifically sought medical care out of a pursuit for certainty, such as consulting another doctor if symptoms persisted despite reassurance from previous doctors, as a preventative method to avoid future illness, or due to a lack of initial support from family members in addressing their health concerns. In addition, Halldorsson and Salkovskis (2017) qualitatively explored excessive reassurance seeking among participants with obsessive‐compulsive disorder or health anxiety (using a screening questionnaire to assess DSM‐IV hypochondriasis criteria). The authors found that among individuals with health anxiety, reassurance seeking was often reported to be driven by intrusive health‐related thoughts, misinterpretation of bodily symptoms, and a fear that something will go wrong with their health. Furthermore, the authors identified several factors that were reported by health‐anxious individuals as motivators to seek reassurance. These included a need for safety due to fear of a fatal diagnosis or death, a desire to reduce anxiety, or to ease the pressure of feeling solely responsible by sharing responsibility for their health. However, excessive reassurance seeking in this study was not limited to medical care, it also included self‐reassurance, as well as reassurance seeking from friends, family, and sources such as the internet and books. However, a gap remains in understanding whether these motivations and findings from these studies apply to individuals diagnosed with IAD, particularly those in the ‘care‐seeking’ subtype, as well as why some individuals alternate between seeking and avoiding care.

To our knowledge, there has not yet been an empirical exploration into why individuals with IAD seek and avoid medical care. The aim of our study was to investigate these behaviours in people who met diagnostic criteria for IAD using a reflexive thematic analysis of in‐depth interviews. Given that past research in our lab has found that fluctuating between seeking and avoiding care is most prevalent in IAD (Newby et al., 2017), we were also interested in understanding what factors or conditions lead an individual to alternate between these behaviours. As this study is exploratory in that it seeks to understand experiences when seeking and avoiding care, no hypotheses were made. For our methodology, we selected a reflexive thematic analysis approach because it allowed us to gain a deeper understanding of participants' experiences from their subjective viewpoints and to analyze patterns and themes within the data, providing valuable insights into our research question.

## METHOD

### Participants and procedure

The research study took place between November 2022 and 2023. Participants were recruited via online social media advertisements (i.e., Instagram and Facebook), targeting individuals who self‐identified as having health anxiety or were worried about their health. Interested individuals were directed to an online form developed in Qualtrics, which included an eligibility questionnaire, an online participant information statement, and a consent form. Individuals over the age of 18 were eligible if they were (1) Australian residents, (2) fluent in English, and (3) self‐identified as experiencing persistent and frequent concerns and worries about their health.

Following the consent process, participants completed a 45‐minute survey, which included demographic questions and self‐report questionnaires to characterize the sample (see details below). Upon survey completion, participants were contacted by a researcher via phone to organize a time and date to complete the qualitative and diagnostic interview using the Anxiety Disorders Interview Schedule (ADIS‐V; Brown & Barlow, 2014), which was conducted over Zoom Videoconferencing (2016) by a researcher trained in the administration of the ADIS or one of the two qualified clinical psychologists and researchers. The purpose of the diagnostic interview was to assess whether participants met current diagnostic criteria for DSM‐5 IAD and other comorbidities (i.e., somatic symptom disorder, generalized anxiety disorder, major depressive disorder, agoraphobia, panic disorder, and obsessive‐compulsive disorder).

If participants met IAD criteria, they were asked open‐ended questions about their experiences and reasons for seeking and avoiding medical care. If participants did not meet IAD criteria, the interview concluded. Participants were reimbursed with an electronic gift card at a rate of $35/h at the completion of the interview for their time completing both the survey and interview. The University of New South Wales Human Research Ethics Committee (HC220649) approved the study, and all participants provided electronic informed consent to participate.

### Measures and materials

#### Online survey

Data collected via the online survey included demographic information (age, gender, residence in Australia, relationship and employment status, and level of education), current mental health treatment, and a battery of self‐report symptom severity measures used to characterize the sample.

The following battery of symptom severity measures was used in the present study and has been validated in previous literature: The Short Health Anxiety Inventory 18‐item (SHAI‐18; Salkovskis et al., 2002) measured health anxiety severity, the Patient Health Questionnaire 9‐item (PHQ‐9; Kroenke et al., 2001) assessed depression severity, the Patient Health Questionnaire Somatic Symptom Severity Scale 15‐item (PHQ‐15; Kroenke et al., 2002) measured the severity of somatic symptoms, and the 7‐item Generalized Anxiety Disorder Scale (GAD‐7; Spitzer et al., 2006) assessed levels of generalized anxiety. Participants were also asked to self‐report the help‐seeking behaviour that best described them from the following options: care‐seeking (i.e., ‘I frequently seek assessment/treatment/medical care for my health concerns’), care‐avoidant (i.e., ‘I tend to avoid medical health care’), and care‐fluctuating (i.e., ‘I fluctuate between avoiding and seeking medical health care’).

#### Diagnostic and qualitative interviews

The interviews were conducted by the three researchers through Zoom Videoconferencing (2016) and ranged between 19 and 60 mins. An abbreviated version of the Anxiety Disorders Interview Schedule for DSM‐5 Adult Version (ADIS‐5; Brown & Barlow, 2014) assessed current IAD and comorbidities. IAD was not required to be the principal psychiatric diagnosis.

Qualitative interviews were delivered in a semi‐structured format, and discussions were prompted using open‐ended questions. The research team collaboratively developed the open‐ended questions to address the aims of the study (see Supplementary File for all interview questions). Examples of the types of questions included were: ‘What generally prompts you to seek medical care?’, ‘What were you hoping to find?’, ‘What generally stops you from seeking care?’, ‘For you personally, how do you find your experiences with health professionals?’, ‘In general, what stages of your life have you avoided health professionals, settings or examinations and why?’ The interviews were initially transcribed by Zoom Videoconferencing (2016) and later checked for accuracy by two researchers.

### Data analysis, quality, and rigour

In preparation for data analysis, zoom transcripts were checked against the audio recordings for accuracy by two researchers, participant information was de‐identified and replaced with numbers, and any identifying information (i.e., personal address, place of work) was removed. Data has been reported using ‘Standards for Reporting Qualitative Research’ (O'Brien et al., 2014) and analyzed in Microsoft Excel (2024) using Braun and Clarke's six‐step ‘Reflexive Thematic Analysis’ process (Braun & Clarke, 2019). This involved: (1) familiarization with the transcripts and interviews; (2) generating initial codes (i.e., short label/phase that captures a meaningful idea within the data); (3) searching for themes and subthemes within the codes; (4) reviewing themes; (5) defining and naming themes; (6) writing up themes. The research team selected these methods given their flexibility in analysis and addressing the exploratory research questions about help‐seeking and avoidance.

The initial phase of the analysis involved familiarization with the transcripts and correcting spelling and grammatical errors. A non‐clinician researcher and clinical psychologist/researcher independently coded five transcripts using a top‐down, general deductive approach, guided by the research question about experiences seeking and avoiding medical care. These initial codes were used to develop a preliminary coding framework, which involved organizing and labelling the codes to structure the data in the early stages of analysis. Then, the researchers collaboratively revised their coding frame and made updates to the frame through discussion with a third rater. The revised coding frame was used to double‐code the remaining transcripts by the initial coder and another clinical psychologist/researcher. Any disagreements were resolved through discussion with a member of the research team. Two members of the research team and previous coders iteratively conducted the generation of higher‐order themes and subthemes to ensure they remained distinctive and clearly defined. Final themes and subthemes are presented in the results.

#### Reflexivity

The research team was comprised of four clinical psychologists/researchers and one non‐clinician researcher. Two members of the team had previous experience conducting a reflexive thematic analysis. The four clinicians had experience working with people with health anxiety symptomology, whereas the non‐clinician researcher did not but had an interest in health anxiety and contributed a fresh and curious perspective. We acknowledge that our past clinical experiences working with health anxiety or familiarity with the literature could lead to biases and assumptions, which could have inevitably influenced the analysis of the interview data. To minimize this, we regularly met to discuss the data to ensure transparency and that our themes and subthemes were grounded in data.

## RESULTS

A total of 386 individuals consented to the study, with 262 completing the survey. These participants were invited to complete the interview, and 118 successfully did so. However, 144 were non‐contactable or missed their scheduled interview time (see Figure S1 in Supplementary File for participant flow and attrition). During the diagnostic interview, 39 participants met DSM‐5 criteria for IAD. However, two participants experienced technical difficulties with Zoom, resulting in their interviews not being recorded or included in the analysis. The final sample consisted of 37 participants.

### Sample characteristics

The characteristics of the sample can be found in Table 1. The mean age was 39 years (range 21–67; SD = 10.8). The majority of the sample were female (75.7%), of Australian ethnicity (84.4%), residing in major cities in Australia (73%), married or in a de facto relationship (62.2%), employed full‐time (40.5%) or part‐time (35.1%), and had completed a university undergraduate or postgraduate degree (62.1%). Just over half were currently receiving treatment for their mental health (54.1%), with the majority receiving treatment in the form of medication (55.6%) or therapy with a psychologist (48.1%). Approximately 32.4% of the sample had no other DSM‐5 mental health comorbidities that were assessed. Of those with comorbid disorders, comorbid generalized anxiety disorder (45.7%) or obsessive‐compulsive disorder (32.4%) were most common. As shown in Table 1, the mean scores on the SHAI‐18 were in the clinical range, and average scores on the PHQ‐9, GAD‐7, and PHQ‐15 were in the moderately severe range. Most participants self‐reported as care‐fluctuating (67.6%), followed by care‐seeking (27%) and care‐avoidant (5.4%).

**TABLE 1:** Sample characteristics.

Demographic information (*N* = 37)	n (%)
Gender	
Man or male	7 (18.9)
Woman or female	28 (75.7)
Non‐binary or a different term	2 (5.4)
Ethnicity	
Australian	27 (84.4)
Other	10 (15.7)
Geographical location in Australia	
Major cities/urban	27 (73)
Regional or remote	10 (27)
Relationship status	
Single	10 (27)
De facto/married	23 (62.2)
Divorced/separated/widowed	4 (10.8)
Employment status	
Unemployed	7 (18.9)
Employed full‐time	15 (40.5)
Employed part‐time	13 (35.1)
Stay‐at‐home parent	3 (8.1)
Carer for a family member (not children)	1 (2.7)
Student	2 (5.4)
Level of education	
High school level	3 (8.1)
Certificate/diploma	11 (29.7)
University undergraduate degree	13 (35.1)
University postgraduate degree	10 (27)
Currently in mental health treatment	20 (54.1)
Current treatment type	
Medication	15 (55.6)
Therapy with a psychologist	13 (48.1)
Therapy with a psychiatrist	1 (3.7)
Support from GP	9 (33.3)
Counselling mental health professional (i.e., nurse, social worker)	4 (14.8)
Over‐the‐counter medication (e.g., vitamins)	7 (25.9)
Comorbidities with other DSM‐5 diagnoses	
Generalised anxiety disorder	16 (45.7)
Panic disorder	3 (8.1)
Agoraphobia	8 (21.6)
Obsessive‐compulsive disorder	12 (32.4)
Major depressive disorder	10 (27)
Somatic Symptom Disorder	11 (29.7)
No comorbidities with listed DSM‐5 diagnoses	12 (32.4)
Symptom severity	M (SD)
Health anxiety (SHAI‐18)	31.8 (6.5) clinical range
Somatic symptoms (PHQ‐15)	13.5 (4.4) moderate range
Depression (PHQ‐9)	11.5 (6.1) moderate range
Generalised anxiety (GAD‐7)	10.9 (1.2) moderate range

### Interview findings

The analysis generated four distinctive themes: 1) Reasons for care‐seeking, 2) Reasons for care‐avoiding, 3) Reasons for fluctuating between seeking and avoiding care, and 4) Experiences with health professionals that impact help‐seeking. These themes and subthemes reflect participants' perspectives and subjective experiences of seeking and avoiding medical care. Themes, subthemes, and illustrative quotes are summarized below and accompanied by Table 2.

**TABLE 2:** Themes, subthemes, and illustrative quotes.

Theme 1: Reasons for care‐seeking	
Subthemes	Quote
Physical symptoms prompt care	Physical symptoms would probably make me go seek medical care I guess something would have to be wrong or something would have to hurt, even if it's a mild sensation, even if it doesn't go into something serious. I consider it kind of proactive on my part to kind of… to address something before it grows into a bigger sensation or a bigger pain
Need for reassurance	I guess for them to do a test to reassure me that it is nothing But I do sometimes request it for peace of mind as well and reassurance To verify my worries about my health because I'm looking for some sort of diagnosis
Previous negative experiences led to a search for better care	So I pretty much take the approach. If they're not the right person. I'll find someone else I think I saw nine doctors before I got to the one that I was happy with…
Ability to care for children	Ability to care for my children. So most of the time I will just suck it up and get on with it. But if I see that it's starting to impact in my parenting physically When I was being pregnant, having young children, I use them more because I had to get more frequent check‐ups and things like that and it's not just me, it's another child
Worry prompts care	So if something is worrying me at night, like it's really becoming an obsessive thought, then I'll go and see someone about it If something is worrying me a bit more than, I'll schedule a routine appointment for a prescription that I'll go and ask

Theme 2: Reasons for avoiding care	
Subthemes	Quotes
Financial reasons	Like for my eyes, for example, I'm not getting treatment. I'd put off getting treatment for that because it was going to be expensive And a cost thing as well, because I don't have a job. I'd have to go pay to go to the doctor and if it was just a, “Everything's cool, fine, go home,” then you've wasted your $100 or whatever it is now to go see a doctor
Fear of being diagnosed with a feared illness	But I don't want to go to the doctor because they're going to just confirm my worst fears I don't want to know the answer, I don't want to be told
Receiving care increases anxiety	I think just because of the anxiety, it started to heighten and then I started to avoid going For an x‐ray for example I'll spend a week worrying about, you know, has the x‐ray given me cancer and just the whole thing I just avoid it
Busy lifestyle	I've got to get a filling in my tooth which I've been probably putting it off for 8 months. I've got to go do that, but that's more so a time thing just going haven't gotten the chance
Feelings of not being taken seriously	So I guess that's a non‐trust thing. I don't know that I can really trust them to know anyway, so why?’ Oh, it's probably just sometimes it's actually maybe deep down in the back of my mind… I know that what I'm saying is silly and that I'm going to say this out loud to someone, and they're going to be like you being ridiculous like’
Inevitable negative outcome	It's silly, but I almost feel like it's gonna happen, whatever happens, happens and I don't have control over it basically’ like the writings on the wall, I feel sometimes and just, you know, can you really avoid sh*t, or it's just gonna happen
Generally avoidant	I avoid a lot of things that aggravate my anxiety. So I avoid going in cars at all, or I avoid going outside when there's a chance of thunderstorm or heavy rain I avoid doing any presentations or doing things in front of people or sometimes just talking to people. I used to avoid talking on the phone a lot

Theme 3: Reasons for fluctuating between seeking and avoiding medical care	
Subthemes	Quotes
Care‐seeking patterns based on physical symptoms	It's gotta be a pretty big. It's gotta be a symptom. It's gotta be a pretty nagging symptom I think if I'm very sick I'll go, but if I think I'm mildly sick, I won't I mean minor stuff, I probably would just go to the doctor if I had to, but anything that would require like a hospital visit or long‐term therapy, or something you know a chronic illness or something severe like cancer I just think I wouldn't cope
Emotionally fatigued from seeking care, so avoids it	I do have a pattern of, oh, this couple of months I'm having a lot of appointments. After a while, I feel tired, and I probably won't do anything for six months and then the cycle starts again I just get a shit ton of advice, like a list of all things I've got to change in my life, and then I get overwhelmed and just don't do it anyway
Worry about missing serious disease(s) prompts care‐seeking	And there'll be a point where I go… I don't know what make what tips the balance, but there is a point we're like, oh, I really have to do this because the risk of leaving it is worse And I get anxious that maybe you know something… because I didn't go on time. They'll… I'll miss something, and that will lead to more issues, so yeah

Theme 4: Experiences with health professionals that impact help‐seeking	
Subthemes	Quotes
Positive experiences with health professionals	Look they're helpful. They are willing to listen as a general rule of thumb, they have good attitudes to helping me and drive good outcomes and yeah, overall it's been a positive experience I did recently see a really good doctor, who was a woman's health doctor, and she really seems, you know, she really was listening to me. I just and I really had a good experience with her
Dismissed and invalidated experience	Yeah, and like gaslighting you into believing that you not, like, there's not something wrong I'm just afraid of that happening to me or somewhere else again, where the doctors are just going to ignore symptoms and dismiss everything I was made to feel as if I was being very anxious and a hypochondriac, and stupid for having come in with those symptoms
Lack of understanding of health anxiety	I haven't come across a lot of health professionals who really understand health anxiety I'm surprised, but many doctors don't know how to manage health anxiety, and that not many psychologists know how to manage it
Want to feel part of their own decision‐making and care	I would like them to treat me like I am the expert in my body and my lived experience Sometimes they don't keep me informed about my own healthcare, which I don't like
Poor interpersonal skills	If you've spent any time in the ICUs or in hospitals, you know, that these people is what they do, and their bedside manners is not the best So I can be quite disappointed sometimes with doctors because a lot of doctors have really poor social skills
Worry about being judged and rejected by doctors	Because I'm feeling silly. Yeah, I don't… because I fear that he might go, “What are you doing?” You know, “Why you asking for another test, and all that?” That would be that. But that's the anxiety part of me ticking away, so… I feel like, if I did open up that everything, I was worrying about maybe they wouldn't want to see me anymore I just I think it was more just me thinking I'm wasting their time sort of. So…. that was my probably my concern. It's not anything, anyone said or did

#### Theme 1: Reasons for care‐seeking

This theme reflects participants' reasons for seeking medical care, including visiting doctors, specialists, or undergoing medical tests.

##### Subthemes

- *Physical symptoms prompt care*: Participants reported that a physical symptom or sensation would motivate them to seek medical attention. For example,

I guess something would have to be wrong or something would have to hurt, even if it's a mild sensation, even if it doesn't go into something serious. I consider it kind of proactive on my part to kind of… to address something before it grows into a bigger sensation or a bigger pain.

ii *Need for reassurance*: Many participants stated they sought medical care for reassurance that they did not have the disease or illness they were worried about.

But I do sometimes request it for peace of mind as well and reassurance.

Some participants were looking for a diagnosis or confirmation that they had the condition they were concerned about, or an explanation for their physical symptoms.

To verify my worries about my health because I'm looking for some sort of diagnosis.

iii *Previous negative experiences led to the search for better care*: Some participants sought medical care due to dissatisfaction with their current health professional. Reasons included past negative experiences, feeling dismissed or not taken seriously, dissatisfaction with the health professional's competence in conducting medical examinations, and/or a perceived lack of interpersonal skills.

I had really negative experiences with some GPs, and so I just wouldn't go back to them, I would look for an alternative.

iv *Ability to care for children*: A few participants expressed their motivation to ensure they were in the best health to care for their children.

Ability to care for my children. So most of the time I will just suck it up and get on with it. But if I see that it's starting to impact in my parenting physically.

v *Worry prompts care*: Several participants highlighted that health‐related worries typically prompt them to seek medical care.

So if something is worrying me at night, like it's really becoming an obsessive thought, then I'll go and see someone about it.

#### Theme 2: Reasons for avoiding care

In this theme, participants identified the reasons why they decided to avoid medical care and/or settings.

- *Financial reasons*: The high cost of health care was a reason for avoiding medical care, particularly for expensive treatments or when they were unsure if an appointment was necessary or not.
  And a cost thing as well, because I don't have a job. I'd have to go pay to go to the doctor and if it was just a, “Everything's cool, fine, go home,” then you've wasted your $100 or whatever it is now to go see a doctor.
- *Fear of being diagnosed with a feared illness*: The most common reason why participants avoided care was due to a fear of receiving a diagnosis for a serious medical condition.

But I don't want to go to the doctor because they're going to just confirm my worst fears.

iii *Receiving care increases anxiety*: Many participants expressed that receiving care, especially waiting for a test result, appointment, or scan, was anxiety‐provoking, which led them to avoid seeking care altogether.

I think just because of the anxiety, it started to heighten and then I started to avoid going.

iv *Busy lifestyle*: Some participants avoided care due to other commitments in their lives, which included travel, work, and the impact of COVID‐19.
  I've got to get a filling in my tooth which I've been probably putting it off for 8 months. I've got to go do that, but that's more so a time thing just going haven't gotten the chance.
v *Feelings of not being taken seriously*: Some participants indicated that their avoidance was due to a concern about not being taken seriously by health professionals or because they lacked trust in them.
  So I guess that's a non‐trust thing. I don't know that I can really trust them to know anyway, so why?’
vi *Inevitable negative outcome*: Some participants felt that seeking care would not change the outcome of their physical health, leading them to avoid care.

like the writing's on the wall, I feel sometimes and just, you know, can you really avoid sh*t, or it's just gonna happen.

vii *Generally avoidant*: A few participants explained that their avoidance extended beyond health care and was characteristic of other aspects of their life, including their employment, ability to go out, and public transport.

Avoidance is my go‐to tactic… What you described is me, like, if I can avoid it, I will.

A few specifically mentioned being avoidant in social settings:

I avoid doing any presentations or doing things in front of people or sometimes just talking to people.

#### Theme 3: Reasons for fluctuating between seeking and avoiding medical care

This theme provides insight into why some participants with illness anxiety move from seeking to avoiding care, and vice versa.

- *Care‐seeking patterns based on physical symptoms*: Participants displayed varying care‐seeking behaviours based on their perceived severity of symptoms. Some sought medical care only when experiencing severe and debilitating symptoms.

I think if I'm very sick I'll go, but if I think I'm mildly sick, I won't.

In contrast, others sought care for conditions they perceived as minor, like a cold or flu, but avoided seeking care for more serious conditions, such as cancer.

I mean minor stuff, I probably would just go to the doctor if I had to, but anything that would require like a hospital visit or long‐term therapy, or something you know a chronic illness or something severe like cancer I just think I wouldn't cope.

ii *Emotionally fatigued from seeking care, so avoids it*: Participants reported that seeking care previously has been overwhelming, anxiety‐provoking, stressful, and tiring at times, leading them to avoid it altogether.

I do have a pattern of, oh, this couple of months I'm having a lot of appointments. After a while, I feel tired, and I probably won't do anything for six months and then the cycle starts again.

iii *Worry about missing serious disease(s) prompts care‐seeking*: Some participants reported they usually avoid seeking care, but as time passes, they become concerned about the potential risk of a negative health outcome that might occur if they do not seek care, prompting them to seek care.

And there'll be a point where I go… I don't know what tips the balance, but there is a point we're like, oh, I really have to do this because the risk of leaving it is worse.

#### Theme 4: Experiences with health professionals that impact help‐seeking

This theme captures the various experiences people with illness anxiety have had with health professionals in the past and currently. Experiences reported in this theme are likely to have an impact on whether people with illness anxiety seek or avoid care.

- *Positive experiences with health professionals*: Many participants had positive experiences with their current or past medical professionals, highlighting their ability to listen, help, and have a positive attitude.

Look they're helpful. They are willing to listen as a general rule of thumb, they have good attitudes to helping me and drive good outcomes and yeah, overall it's been a positive experience.

However, these same participants also had negative experiences, as outlined in the subthemes below.

ii *Dismissed and invalidated experience*: Most participants described feeling dismissed, invalidated, and not taken seriously by health professionals at some stage. Some felt unheard or as if they were wasting their health professionals' time. Additionally, some emphasized that they felt invalidated when their health professionals attributed their physical symptoms or concerns to health anxiety or ‘being a hypochondriac’.

I was made to feel as if I was being very anxious and a hypochondriac, and stupid for having come in with those symptoms.

iii *Lack of understanding of health anxiety*: A few participants noted that the doctors and psychologists they encountered lacked familiarity with health anxiety or struggled to manage it effectively.

I'm surprised, but many doctors don't know how to manage health anxiety, and… not many psychologists know how to manage it.

iv *Want to feel part of their own decision making and care*: Participants highlighted the importance of a collaborative approach when interacting with health professionals, ensuring there is shared decision‐making and autonomy over their health and treatment.

I would like them to treat me like I am the expert in my body and my lived experience.

v *Poor interpersonal skills*: Some participants found it challenging to communicate with health professionals, as they perceived their bedside manner as lacking.

So I can be quite disappointed sometimes with doctors because a lot of doctors have really poor social skills.

vi *Worry about being judged and rejected by doctors*: A few participants were concerned about being judged by doctors if they shared their health concerns openly.

I feel like, if I did open up about everything, I was worrying about… maybe they wouldn't want to see me anymore.

## DISCUSSION

Although IAD care‐seeking and care‐avoidant subtypes were proposed in the DSM‐5 over a decade ago, no previous research has explored the experiences of individuals with IAD when engaging in these different patterns of behaviours. This study is the first to describe the experiences of individuals with IAD when seeking and avoiding medical care. Our results showed that the factors influencing individuals with IAD to seek or avoid medical care are complex and multifaceted. The reasons are not simply due to reassurance‐seeking and avoidance due to fears of medical diagnosis. Specifically, our results suggest that both internal affective factors (e.g., worry) and external factors (e.g., cost of care), along with past, often negative experiences with health professionals, influence whether people with IAD seek or avoid medical care.

The current study identified five factors that prompt or influence individuals with IAD to seek medical care. These were internal factors, such as the need for reassurance, health‐related worry, physical symptoms, a motivation to stay healthy for their children, as well as external factors, such as past dissatisfaction with health professionals, which drove the need to seek new health professionals. In contrast to previous research with individuals diagnosed with hypochondriasis (Okita et al., 2016), which found that medical reassurance‐seeking stemmed from dissatisfaction with family support and a desire to prevent disease, participants in the current study described seeking reassurance to obtain an explanation for physical symptoms or to confirm the absence of disease. However, these findings are consistent with those of Halldorsson and Salkovskis (2017), who found that reassurance seeking was triggered by a misinterpretation of physical symptoms and motivated by a need for safety due to fear of being diagnosed with a fatal illness. They also align with mainstream cognitive behavioural theories of health anxiety, which suggest that individuals seek reassurance to reduce their health‐related concerns (Abramowitz & Moore, 2007; Hedman‐Lagerlöf, 2019; Meyer et al., 2023; Warwick & Salkovskis, 1990). However, the discrepancies between the current study and the study by Okita et al. (2016) may be due to differences in diagnostic criteria, as hypochondriasis is considered a much narrower definition of health anxiety compared to IAD (Bailer et al., 2016; Newby et al., 2017). The discrepancy may also stem from differences in the samples used (primary care sample vs. a sample recruited via online social media advertisements in the current study), the possibility of sampling bias and variability inherent in studies of this nature, or due to the nature of the questions asked in the hypochondriasis study, which focused on why individuals sought reassurance specifically, rather than exploring broader reasons for seeking medical care. Our novel findings into the reasons for care‐seeking suggest a complex interplay between internal and external influences. More research is needed to understand which factors play the most significant role and are most predictive of health care utilization in the long term.

Our study revealed several factors that influence an individual with IAD to avoid medical care, including internal factors such as a fear of being diagnosed with an illness, concerns about not being taken seriously by health professionals, beliefs around an inevitable negative outcome, anxiety around getting medical exams/tests, and a general tendency towards avoidance behaviours. External factors were also identified, including the cost of medical care and a busy lifestyle. Studies exploring why individuals with IAD avoid care are lacking; therefore, we draw on broader literature from the general population to contextualize our findings. Our findings align with those in the general population, as a lack of time, negative past experiences with health professionals or distrust of doctors, and a fear of having a serious disease or undergoing medical treatments have been identified as barriers to seeking healthcare (Kannan & Veazie, 2014; Taber et al., 2015). However, a belief around an inevitable negative medical outcome leading to care avoidance was a novel finding in the current study, which might be specific to individuals with IAD. These findings align with the single other qualitative study, which found that participants with IAD exhibited a negative orientation towards the future (Brady & Braz, 2023). The cost of health care has been identified as a significant barrier to seeking care (Callander et al., 2017; Corscadden et al., 2017; Smith et al., 2018; Sulku et al., 2023; Walkom et al., 2013), highlighting a large practical barrier that needs to be addressed among both the general population and those with IAD. The findings from this theme suggest that individuals with IAD experience both internal and external drivers that lead them to avoid seeking medical care. Internal factors seem to be motivated by fear and anxiety, whereas external and practical barriers may further influence their avoidance behaviour.

One past study found that most individuals with IAD fluctuate between seeking and avoiding medical care (Newby et al., 2017). Our results indicate that this behaviour may depend on the severity of their physical symptoms, with some seeking care for severe physical symptoms and others for only minor conditions. In addition, we found that help‐seeking can be overwhelming and anxiety‐provoking for some, leading them to avoid care altogether, while others, who typically avoid care, may be driven to seek care by the prolonged fear of missing a serious disease. To our knowledge, there are no studies that qualitatively explore why individuals with or without IAD fluctuate between seeking and avoiding medical care. There is some research from the breast cancer literature that finds an ‘inverted U' with respect to breast cancer worry and health care utilization, specifically mammography use, with high and low levels of worry associated with lower screening use and moderate levels of worry associate with higher screening use (Andersen et al., 2003). However, these patterns have not been investigated in IAD. A future study is needed to examine whether the same ‘inverted U' pattern in health care utilization is present in IAD, and to identify which individual factors predict the tendency to avoid care for severe physical symptoms, while seeking care for less concerning symptoms.

Past experiences with health professionals were an important factor discussed in the interviews, which likely have an important impact on participants' help‐seeking behaviours. While many participants highlighted positive experiences with health professionals, many also reported feeling dismissed or invalidated, or worried about being judged by doctors. Some expressed a desire for autonomy and shared decision‐making in their care. This finding is consistent with previous qualitative research in individuals with IAD (Brady & Braz, 2023), individuals with high health anxiety (Bulut & Bozo, 2022; Le et al., 2022; Thompson, 2020) and individuals with medically unexplained symptoms (Shillaker et al., 2024), all which report challenging experiences with health professionals and difficulties communicating with them. Many participants in the current study also felt that health professionals lacked understanding of health anxiety and had poor interpersonal skills. This perception is noteworthy, as research into general practitioner perspectives' when dealing with patients with medically unexplained symptoms shows that general practitioners feel they lack psychological skills to work with these clients (Salmon et al., 2007). This highlights the need for more knowledge and training for health professionals in assessing and treating their patients with health anxiety in routine care, to ultimately build a positive relationship with their patients and allow them to feel validated and heard.

### Limitations

The current study has a number of limitations. The sample primarily consisted of well‐educated females from major cities in Australia, which may limit the generalizability of the findings to people with health anxiety who have different social, demographic, and clinical characteristics from the current sample. Next, the sampling strategy relied on individuals self‐identifying as health anxious, suggesting that the sample had some awareness of their illness anxiety. This may have influenced the findings, as many individuals with IAD attend medical services rather than psychological services, often perceiving their concerns as purely medical. As such, the conclusions may have differed if the sample had included individuals presenting to medical settings, where awareness of the influence of psychological factors on distress may be lower. It is unknown whether the findings generalize to the broader population of individuals with IAD in the community or people with other mental health disorders or the general population more broadly.

### Conclusions and future directions

The findings from the current study highlight the complexity and multifaceted nature of care‐seeking and avoidant behaviours in IAD. They suggest that IAD is more heterogeneous than the DSM‐5 subtypes imply, and that the DSM‐5 may overlook the nuance that many individuals with IAD fluctuate between seeking and avoiding care. Furthermore, they highlight the importance of ongoing assessment in individuals with IAD, as the factors that influence care‐seeking and avoidance fluctuate for many individuals and may inform differential treatment strategies. Specifically, our findings reveal that multiple psychological and practical factors influence help‐seeking behaviour. Future research should examine whether there are meaningful differences between the subtypes in their demographic and clinical features. This knowledge could help determine whether the subtypes are reliable, valid, and useful, or whether it would be more effective to treat these behaviours as symptoms of IAD and omit the subtype classification. More research is also needed to explore these patterns of behaviour across different contexts, such as primary and secondary medical care settings, and to understand the natural course of who seeks care, when, and for what reasons, as well as who is at risk of overusing or avoiding health care services.

## AUTHOR CONTRIBUTIONS

**Katarina Kikas:** Conceptualization; data curation; formal analysis; investigation; project administration; visualization; writing – review and editing; writing – original draft. **Aliza Werner‐Seidler:** Conceptualization; methodology; writing – review and editing; project administration; resources; supervision; validation; formal analysis. **Brittany Corkish:** Investigation; formal analysis; writing – review and editing. **Emily Upton:** Investigation; writing – review and editing. **Monique Holden:** Writing – review and editing; investigation. **Jill M. Newby:** Conceptualization; methodology; project administration; funding acquisition; resources; supervision; validation; writing – review and editing; formal analysis.

## CONFLICT OF INTEREST STATEMENT

The authors declare no conflicts of interest.

## Supporting information


Data S1.

## Data Availability

The data supporting the current research findings will not be made publicly available on a data repository due to the sensitive nature of the data. However, upon reasonable request to the last author, the data may be shared for research purposes, pending ethical approval.
