# Supplementary material for: Illness anxiety disorder: A qualitative study of people with health anxiety and their experiences seeking and avoiding medical care
Source: Br J Clin Psychol. 2025 Jul 8;65(1):68–85. doi: 10.1111/bjc.70005 (PMC12889205; doi:10.1111/bjc.70005)
Supplement: Supplementary file 1 — Data S1. [file BJC-65-68-s001.docx]

**Supplementary File**

**Nonredacted ethics statement**

The University of New South Wales Human Research Ethics Committee (HC220649) approved the study, and all participants provided electronic informed consent to participate.

**Interview questions**

1. Where do you currently and have previously gone for medical care? Medical care such as getting a test done (x-ray or scan) or visiting a GP?
2. What generally prompts you to seek medical care?
3. What were you hoping to find?
4. How long have you sought care?
5. What are the benefits of visiting health care examinations, health professionals such as doctors or settings such as GP clinics or hospitals for you?
6. Are there any drawbacks?
7. Have you ever avoided medical care? OR (if they mentioned they avoid care) You mentioned you avoided care when... can you tell me what generally stops from seeking medical care?
8. How long have you avoided care?
9. What are the benefits of avoiding health care examinations, health professionals such as doctors or settings such as GP clinics or hospitals for you?
10. Are there any drawbacks?
11. In general, what stages of your life have you avoided health professionals, settings or examinations/tests and why?
12. What other experiences or difficult situations have you had that you generally avoided?
13. For you personally, how do you find your experiences with medical professionals? What were you hoping from the medical professional/s?
14. Do you have anything else you want us to know, or want to share?

**Figure 1.** Flow-chart of participants

Consented to the study (n = 386)

Completed the full baseline survey (n = 262)

Completed the diagnostic and qualitative interview (n = 118)

Did not complete full baseline survey (n=124)

Unable to contact (n = 123)

Missed scheduled interview time (n = 21)

Met criteria for IAD (n = 39)

Met SSD criteria (n = 47)

Experienced subthreshold health anxiety (n = 32)
